# A semi-automated technique for labeling and counting of apoptosing retinal cells

**DOI:** 10.1186/1471-2105-15-169

**Published:** 2014-06-05

**Authors:** Mukhtar Bizrah, Steve C Dakin, Li Guo, Farzana Rahman, Miles Parnell, Eduardo Normando, Shereen Nizari, Benjamin Davis, Ahmed Younis, M Francesca Cordeiro

**Affiliations:** 1 Glaucoma and Retinal Neurodegeneration Group, UCL Institute of Ophthalmology, London, UK; 2 The Hillingdon Hospitals NHS Foundation Trust and Western Eye Hospital, Imperial College Healthcare NHS Trust, London, UK; 3 Deparment of Visual Neuroscience, UCL Institute of Ophthalmology, London, UK; 4 NIHR Biomedical Research Centre at Moorfields Eye Hospital, London, UK; 5 Western Eye Hospital, Imperial College Healthcare NHS Trust, London, UK; 6 St George’s, University of London, London, UK

**Keywords:** Automated analysis, Cell counting, Cell quantification, Blob analysis, Image processing, Image analysis, Retinal ganglion cells, Retinal cell apoptosis, DARC, Glaucoma

## Abstract

**Background:**

Retinal ganglion cell (RGC) loss is one of the earliest and most important cellular changes in glaucoma. The DARC (Detection of Apoptosing Retinal Cells) technology enables *in vivo* real-time non-invasive imaging of single apoptosing retinal cells in animal models of glaucoma and Alzheimer’s disease. To date, apoptosing RGCs imaged using DARC have been counted manually. This is time-consuming, labour-intensive, vulnerable to bias, and has considerable inter- and intra-operator variability.

**Results:**

A semi-automated algorithm was developed which enabled automated identification of apoptosing RGCs labeled with fluorescent Annexin-5 on DARC images. Automated analysis included a pre-processing stage involving local-luminance and local-contrast “gain control”, a “blob analysis” step to differentiate between cells, vessels and noise, and a method to exclude non-cell structures using specific combined ‘size’ and ‘aspect’ ratio criteria. Apoptosing retinal cells were counted by 3 masked operators, generating ‘Gold-standard’ mean manual cell counts, and were also counted using the newly developed automated algorithm. Comparison between automated cell counts and the mean manual cell counts on 66 DARC images showed significant correlation between the two methods (Pearson’s correlation coefficient 0.978 (p < 0.001), R Squared = 0.956. The Intraclass correlation coefficient was 0.986 (95% CI 0.977-0.991, p < 0.001), and Cronbach’s alpha measure of consistency = 0.986, confirming excellent correlation and consistency. No significant difference (*p* = 0.922, 95% CI: −5.53 to 6.10) was detected between the cell counts of the two methods.

**Conclusions:**

The novel automated algorithm enabled accurate quantification of apoptosing RGCs that is highly comparable to manual counting, and appears to minimise operator-bias, whilst being both fast and reproducible. This may prove to be a valuable method of quantifying apoptosing retinal cells, with particular relevance to translation in the clinic, where a Phase I clinical trial of DARC in glaucoma patients is due to start shortly.

## Background

Glaucoma is a chronic degenerative optic neuropathy that results in irreversible loss of retinal ganglion cells (RGC; the neurons that relay information from the retina to the cortex). RGC loss, coupled with degeneration of the RGC axons, results in optic disc “cupping” and a progressive visual field loss that is characteristic of glaucoma [1]. In glaucoma, most RGC loss occurs through the process of apoptosis (programmed cell death) [2]. Apoptosis has a central role in several other neurodegenerative diseases [3-5], as well as glaucoma, with evidence that the targeting of pro-apoptotic activity may be neuroprotective against Neurodegeneration [3-10].

Glaucoma is often diagnosed late in the course of the disease using the gold standard method of perimetry, since visual field defects are not detected until up to 40% of RGCs have been lost [11]. However, since timely intervention can halt (but not reverse) glaucomatous progression, much recent research has focused on identifying early diagnostic markers of glaucoma. RGC apoptosis has been shown to be one of the initial pathological processes in glaucoma [12,13], and its detection could facilitate early diagnosis and management of this condition. One of the first events in apoptosis is externalisation of phosphatidylserine (a membrane phospholipid) from the inner to the outer leaflet of the cell membrane. Annexin V is a protein with a high affinity to exposed phosphatidylserine [14]. Imaging of radiolabeled Annexin V therefore enables detection of apoptotic cells. Clinical studies have utilized Technetium-99 m radiolabeled Annexin V for the non-invasive detection and serial imaging of apoptosis in various clinical settings, such as acute myocardial ischemia [15], cardiac allograft rejection [16], breast cancer [17] and anti-cancer treatment induced apoptosis [18,19].

Recently, our laboratory has developed a technique by which Annexin V is labeled with a fluorescent marker, which is subsequently intravitreally administered [12]. A 488 nm wavelength argon laser is used to excite the administered annexin V-bound fluorophore, and a photodetector system with a 521-nm cut-off filter enables detection of the fluorescence light emission. The fluorescent retinas are imaged with Confocal laser scanning ophthalmoscopy. This novel technology has enabled the non-invasive *in vivo* real-time visualisation of single retinal cells undergoing apoptosis, and has been given the acronym DARC (Detection of Apoptosing Retinal Cells). [12] DARC has been used in animal models of glaucoma [20] and Alzheimer’s disease [21], highlighting the role of apoptosis in the early stages of both diseases. It has also been studied in the evaluation of neuroprotective strategies in animal models of glaucoma, such as glutamate modulation [22], amyloid-beta targeting therapy [23] and topical Coenzyme Q10 [23,7].

To date, quantitative assessment of RGC apoptosis has been a manual process. The number of apoptosing RGC’s is counted by one or more persons using software such as ImageJ® [24]. Such manual assessment procedures have several disadvantages related to the precision and accuracy of cell counts. In terms of precision, manual quantification involves subjective judgment increasing operator-dependency - especially when images are of low quality – potentially leading to substantial intra- and inter-operator variability. In terms of accuracy, if the operator is not blinded then this technique is potentially vulnerable to *bias*. Furthermore manual quantification is time-consuming and labour-intensive – especially if more than one individual is needed to maximise precision and accuracy – rendering the analysis of a large number of images challenging.

In this study, a semi-automated technique has been developed for the quantification of apoptosing retinal cells on DARC images. A total of 66 DARC images were analysed by a novel automated algorithm and by 3 human operators. The total cell counts of the automated algorithm were compared to the mean cell counts of three human operators. The automated algorithm was found to minimise operator-dependency while providing fast, accurate, and reproducible cell-counts.

## Methods

### DARC images

**Figure 1:**
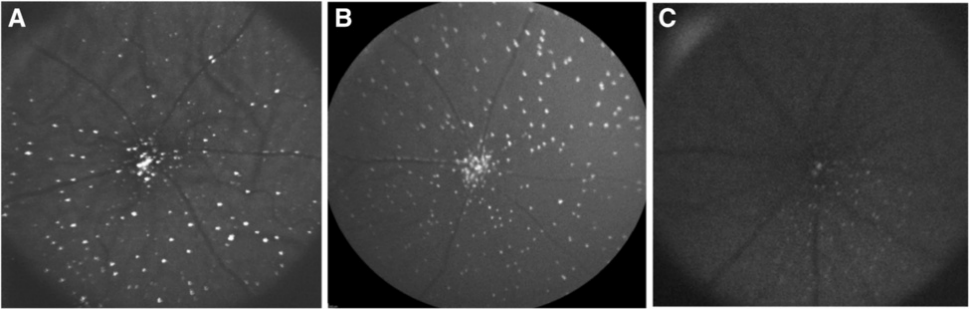
Images A, B & C are examples of DARC images before undergoing manual or automated cell labeling.

DARC images were randomly selected from a database of approximately 3000 DARC images of rat eyes, which had either undergone surgically-induced intraocular pressure (IOP) elevation or had been exposed to neurotoxic substances or various treatments, at different time points. Images were captured as described in previous publications [12,21,20] and operators were blinded to the type of insult which the eyes had undergone. The quality of images spanned a wide range in order to investigate the robustness of the technique. Figure 1 below represents examples of the variation in quality of the DARC images. Note that apoptosing retinal cells, imaged using a confocal laser scanning ophthalmoscope, appear as ‘white spots’ on the retina as previously described [12,20].

### Cropping and re-sizing of DARC images pre-analysis

DARC images were cropped to remove descriptive text at the bottom and to eliminate peripheral noise. They were then re-sized to 600 pixels square using the bilinear interpolation algorithm built into the “image resize” function in Adobe Photoshop (Adobe Inc). This was done purely in order to reduce image-processing time and we see no systematic influence of this level of down-sampling on processing of a random sample of the images tested.

### Manual analysis

Manual image analysis was performed by three blinded operators using ImageJ® (National Institutes of Mental Health, USA) [24]. The ImageJ ‘multi-point’ tool was used to label each structure in the image classed as an apoptosing cell. As each cell is labeled it is assigned a unique number enabling manual quantification of the total number of visible single apoptosing retinal cells an example of a manually labeled DARC image is shown.

### Automated analysis

The Matlab® (Mathworks Ltd) programming environment was used to develop a program for labeling and counting apoptosing retinal cells in DARC images. The stages of the semi-automated analysis performed by the program are described below. Of note, it is possible to automate the cropping and re-sizing of images by adding these functions to the Matlab script. This will enable the image analysis to be fully automated.

#### Stage 1: Pre-processing

A single DARC image can contain wide fluctuations in mean luminance and contrast levels within a given image-region, which can interfere with subsequent thresholding and spatial filtering. To counteract this local luminance and contrast, structure was “flattened” within each image. Specifically, the mean and standard deviation of the grey levels in the locale of a given pixel are computed and used to effectively convert the pixel grey-level into a local *z*-score. To compute statistics within a locale we used convolution with Gaussian spatial filters, i.e. the local mean luminance of a pixel and its locale is simply a Gaussian blurred version of the original:

(1) $$\mu =G_{s}⊗I$$

where *I* is the source image and *G*_*s*_ is a two-dimensional Gaussian filter (standard deviation, *s*). Similarly, the Gaussian-weighted standard deviation can be computed as follows:

(2) $$\sigma =\sqrt{G_{s}⊗I^{2}-\mu^{2}}$$

so that the final pre-processed image is then:

(3) $$Z=\frac{G_{s}-\mu }{\sigma }$$

**Figure 2:**
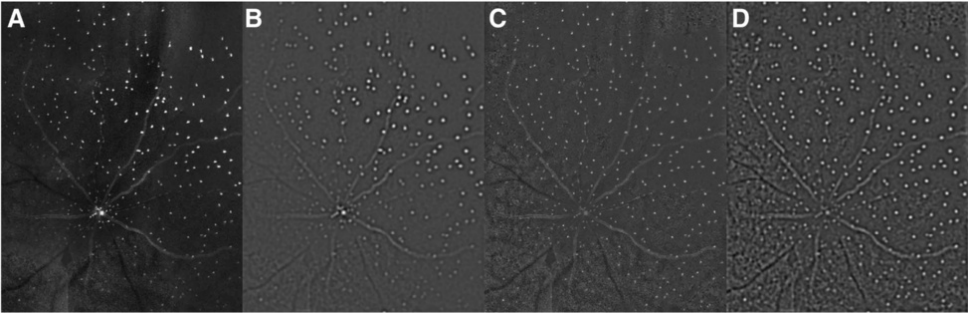
**The effects of pre-processing illustrated on a DARC image. (A)** A raw DARC image. **(B)** The same image filtered with a Δ^2^*G*_*t*_ filter **(C)** A pre-processed version of **(A)** (corrected for local variation in luminance structure). **(D)** A Laplacian filtered version of **(C)**. Compare **(D)** to **(B)** and note presence of additional image structure in **(D)**.

The resultant Z is then processed with a conventional Laplacian-of-Gaussian (Δ^2^*G*_*t*_) spatial- frequency band-pass filter (with standard deviation, *u*) to highlight high-energy isotropic image-structure. The operation of such a filter on DARC images that have and have not been pre-processed is illustrated in Figure 2A-D below.

Figure 2A is the original image and 2B the result of filtering it with the Δ^2^*G*_*t*_ filter. Note the weak (low-contrast) filter-responses in the lower left portion of the image in 2B. Figure 2C shows the pre-processed version of Figure 2A (generated with Eqs 1–3); note the uniformity of luminance and contrast structure therein. Figure 2D is a Laplacian-filtered version of the pre-processed image (Figure 2C). Note that the filter response is now much more spatially uniform than in 2B. The candidate vasculature and cell-structure is now visible across the whole image, and will remain so after global thresholding used to isolate discrete image structure. The parameters used to pre-process the 600 pixel square source images were: *s* = 64 pixels, *u* = 1.5 pixels.

#### Stage 2: Cell identification

**Figure 3:**
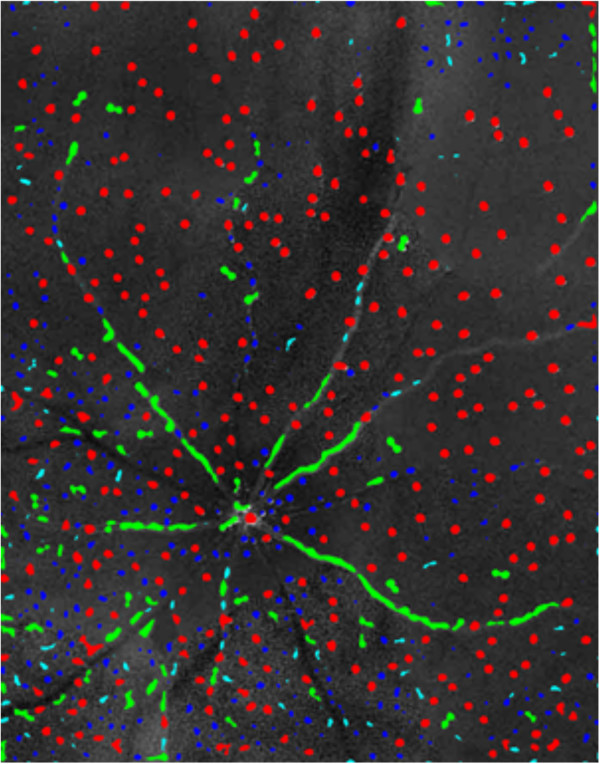
**An illustration of the DARC image shown in Figure **2**d after undergoing thresholding and a novel ‘blob analysis’ stage to classify blobs as cells (red), vessels (green) or noise (blue).**

To identify image structure as cells we first apply image-thresholding to the filtered images; this simply sets all grey levels falling too near to the mean grey level of the whole image, to zero. The threshold (*T*) was fixed at 1.8 × the standard deviation of the image grey-level, which generally gives good subjective delineation of cell and vessel structure in the image. We then employed “blob-analysis” (using the regionprops routine in MatLab®) on the isolated regions that resulted from thresholding. This yields various features of each blob including the length along major (*L*_maj_) and minor (*L*_min_) axes length, its area (*A*) and the location of its centroid ([*C*_x_,*C*_y_]). We next perform categorisation of image structure based on these estimates. In Figure 3, blobs have been categorized as cells (red), vessels (green) or noise (blue), based on the following criteria:

$$\mathrm{For}\;\mathrm{noise}:A<A_{\text{min}},\mathrm{for}\;\mathrm{vessels}:\left(L_{\mathrm{maj}}/L_{\text{min}}\right)>{\mathrm{Aspect}}_{\text{min}},$$

and all other blobs are classed as cells.

Pilot studies were performed to maximize agreement between the automated and manual cell counts (n.b. the inclusion of this stage is why we refer to the technique as ‘semi-automated’ rather than fully automated). Setting *A*_min_ (minimum area - in square pixels- for a blob to be a candidate cell) to 9.0 and *Aspect*_min_ (the minimum aspect ratio for a blob to be a candidate blood vessel) to 3.0 yielded total cell counts which best corresponded with mean manual cell count of three inexperienced and masked operators, and was therefore chosen and fixed for automated quantification. This is an important step as altering these parameters results in different classification of blobs. This is particularly true for the *A*_min_ parameter, as this determines the minimum cut-off size for a blob to be classified as a cell rather than noise. The pilot studies enabled the five Matlab algorithm script parameters (*s*, *u*, *T*, *A*_min_ and *Aspect*_min_) to be fixed at the point of image analysis, enabling fully automated analysis by a single operator.

### Study protocol

**Figure 4:**
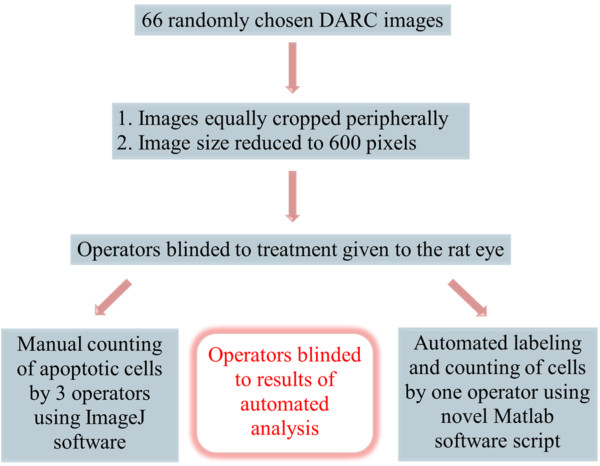
Flowchart summarizing the protocol followed for the manual and automated analysis of the DARC images.

For the purpose of this study, 66 post-insult images were picked randomly from this database with two exclusion criteria: the presence of “white” vessels (thought to be arising from Annexin 5 binding to the vascular endothelium) and insufficient image-quality to support manual cell identification. These images were analyzed using both manual and automated techniques, and this sample-size selected to reflect limits on the operator time available for manual counting. The study protocol is summarized in the flow chart (Figure 4).

As the automated algorithm parameters were fixed, only one operator was needed to perform the automated image analysis.

### Statistical methods

Pearson’s *R*, Intraclass correlation coefficient (ICC) and Cronbach’s Alpha Reliability Coefficient were used to test the correlation, consistency and reliability between manual and automated cell counts. We used Bland-Altman plots to assess the level of agreement between the gold standard (mean manual) cell count and the automated cell count. The paired samples t-test was used to test for a statistically significant difference between manual and automated cell counts.

## Results

### Duration of image analysis

Manual labeling of the cells on an image by a single operator to obtain a total cell count took an average of 3 min ± 2 min (Mean ± 1.96 Standard Deviation). In contrast, generating a labeled image and a total cell count with the automated algorithm took an average of 9 sec ± 2 sec. As all the Matlab script parameters were fixed, the script was only run once on each image.

### Examples of automated labeling

**Figure 5:**
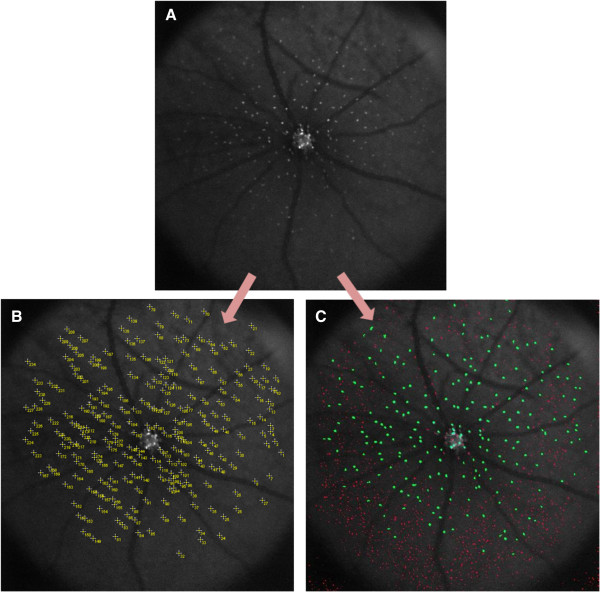
**Example of a DARC image before and after undergoing manual and automated labeling.** Image **A** represents a cropped DARC image before undergoing labeling. Image **B** represents the same DARC image after undergoing manual labeling using ImageJ® ‘multi-point selections’ tool, which marks and numbers each selected spot on the image. Image **C** represents the same DARC image after undergoing automated cell labeling using the novel Matlab® script. In Image **C**, structures identified as ‘cells’ have been labeled in green, whilst ‘non-cellular’ structures have been labeled in red.

Figure 5 below illustrates a DARC image before and after undergoing manual and automated labeling:As shown in Figure 5, manual labeling using the ImageJ® ‘multi-point selections’ tool enables the marking and numbering of each spot on the DARC image (Image Ib). Image Ic represents the same DARC image after undergoing automated cell labeling using the novel Matlab® script. In Image Ic, structures identified by novel Matlab® script as ‘cells’ have been labeled in green, whilst ‘non-cellular’ structures have been labeled in red. The script automatically calculates the total number of spots identified as cells.

### Mean manual cell counts vs automated cell counts analysis

**Figure 6:**
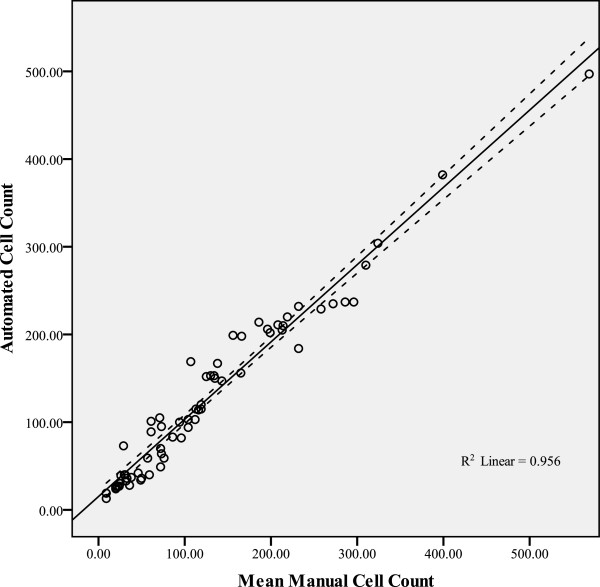
**Correlation between the Mean Manual Cell Counts and the Automated Cell Counts of the 66 DARC images.** The continuous line is the best-fit line, and the adjacent dotted lines represent the 95% confidence intervals.

Pearson’s correlation coefficient for the mean manual cell counts and the automated cell counts was 0.978, p < 0.001 (two-tailed significance). The R squared, as illustrated in Figure 6, was 0.956. The Intraclass correlation coefficient was 0.986 (95% CI 0.977-0.991, p < 0.001). Cronbach’s alpha measure of consistency was 0.986. These results indicate a highly significant correlation and consistency between the mean manual cell counts and the automated cell counts.

In 36 (54.5%) of 66 DARC images, the automated cell count was higher than the mean manual cell count. The mean manual cell count for the 66 DARC images was 125.7 cells, whereas the mean automated cell count was 126.0 cells. The mean automated cell count was therefore 0.23% higher than the mean manual cell count. There was no significant difference between the mean manual and automated cell counts (*p* = 0.922, 95% CI −5.53 to 6.10).

**Figure 7:**
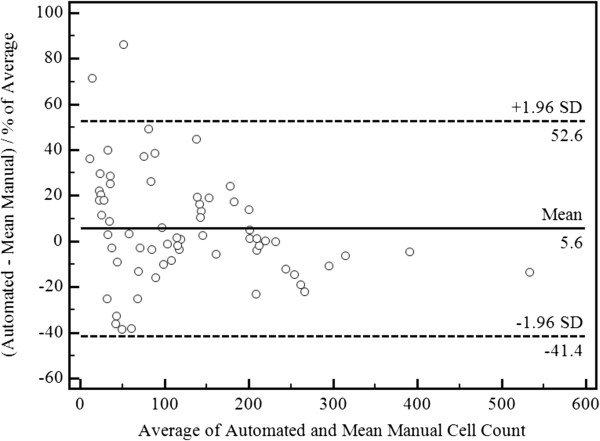
Bland-Altman Plot of Percent Difference of Automated Cell Counts from Mean Manual Cell Counts.

A Bland-Altman ‘percent difference’ plot was constructed as recommended for method comparison studies in which agreement is to be assessed for a wide measurements range [25,26]. As shown in Figure 7, there was strong agreement between the two methods, with 64 (97%) of 66 images cell counts lying within the 95% limits of agreement. The two images lying beyond 1.96 SD from the mean (normally referred to as 95% limits of agreement) are discussed in the next section. There was a tendency for the automated algorithm to underestimate the cell count in DARC images with high cell numbers (>200 cells). As shown in Figure 7, the ratio of the difference of the automated cell counts from the mean manual cell counts was within 1.96 standard deviations of the mean difference for all >200 cell counts. This indicates that the extent of undercounting was minimal. A larger sample size of DARC images with >200 cell counts is needed to assess for a statistically significant difference in automated and mean manual cell counts.

### Cell count differences beyond the 95% limits of agreement

**Figure 8:**
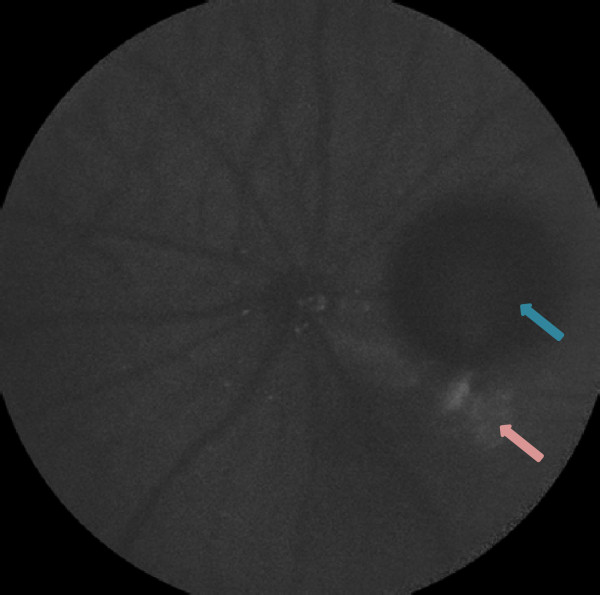
The first DARC image with cell count differences beyond the 95% limits of agreement.

**Figure 9:**
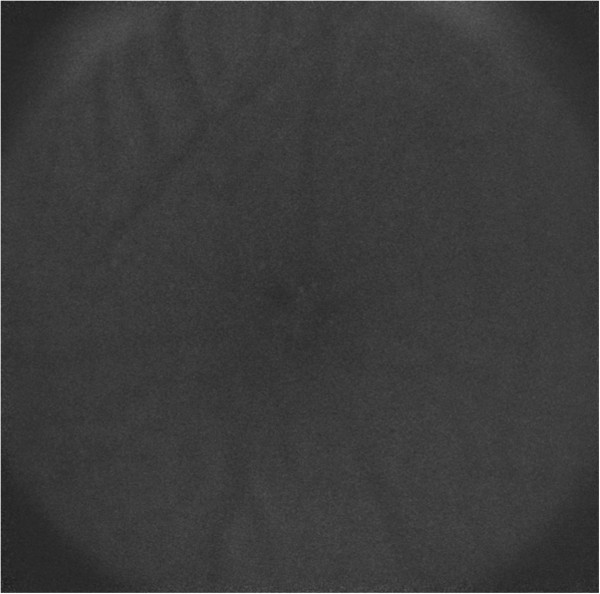
The second DARC image with cell count differences beyond the 95% limits of agreement.

Figure 8 represents a DARC image in which the automated cell count was higher than the mean manual cell count. The image contained non-cellular fluorescent structure (pink arrow) which represents injection artifact, as well as a dark blob (blue arrow) which represents either a bubble (resulting from intravitreal injection) or a haemorrhage. The apoptosing cells in the image exhibited poor fluorescence, making manual cell identification challenging. This is reflected by the large inter-operator variation: The difference between the manual cell counts of operator 1 and operator 2, operator 1 and operator 3, and operator 2 and operator 3 were 32 cells, 41 cells and 9 cells respectively. The mean manual cell count was 29 cells (highest manual cell count = 53 cells), whereas the automated cell count was 73 cells. The higher automated cell count may be due to higher sensitivity of the automated method. On the other hand, the ‘granular’ nature of the retinal background may have resulted in false positive detection of cells.In Figure 9, the cells were very poorly fluorescent, making accurate labeling by an operator difficult. The cell counts for operators 1, 2 and 3 were 21 cells, 0 cells and 7 cells respectively. Hence, while the cell count of operator 1 (=21 cells) was close to the automated cell count (=19 cells), operator 2 did not judge any of the structures to be fluorescent labeled cells. Arguably, such an image with poorly fluorescent cells should not be used to judge the extent of apoptosis, as the manual analysis results are variable and contentious. The higher cell count acquired by the automated technique may be due to higher sensitivity in detecting poorly fluorescent cells, or due to detection of structures which in reality would be not judged as cells because they are not strongly fluorescent. In the presence of such wide variation in manual labeling results, confirmation of the true presence of fluorescent cells is only possible with histological analysis.

### Undercounted DARC images

**Figure 10:**
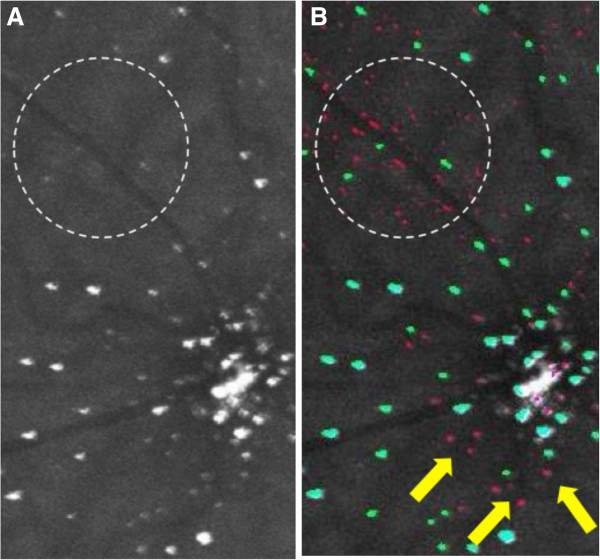
**Example of a cropped and magnified section of a DARC before (A) and after (B) undergoing automated labeling.** Structures identified by the automated algorithm as cells are shown as green spots, whereas spots identified by the algorithm as non-cellular structures are shown as pink spots.

**Figure 11:**
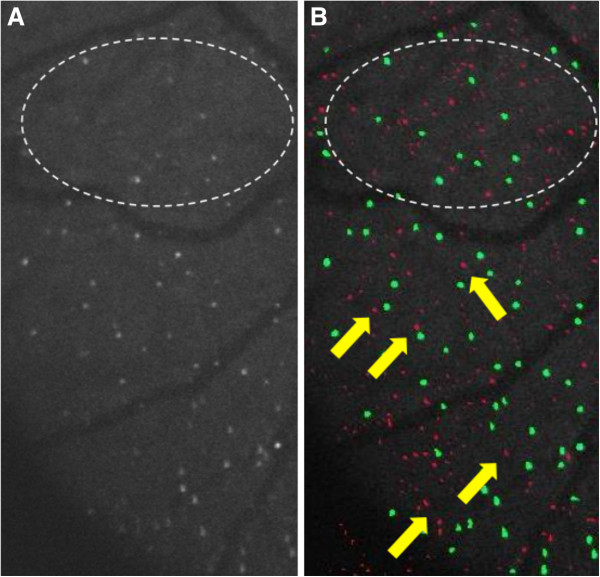
Example of a cropped and magnified section of a DARC image before (A) and after (B) undergoing automated labeling.

**Figure 12:**
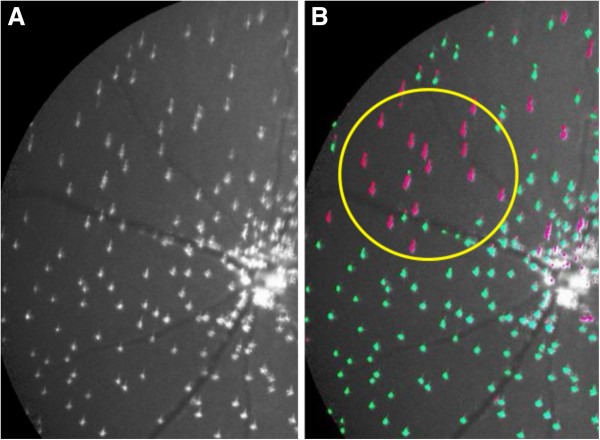
Example of a cropped and magnified section of a DARC before (A) and after (B) undergoing automated labeling.

Figure 10 shows examples of a DARC image with >200 apoptosing RGCs in which cells were undercounted by the automated algorithm.Green labeled spots on Figure 10B represent spots which were labeled and counted as ‘cells’ by the algorithm. Pink spots represent spots labeled as non-cellular structure and therefore not counted as cells by the algorithm. The white circle on the image shows examples of noise correctly identified as such by the algorithm (labeled in pink). On the other hand, the yellow arrows shows spots which should be labeled as cells, but the algorithm in this case has labeled as non-cellular structure (labeled in pink). This is due to the small size and low luminance of these spots. Another example is shown in Figure 11.Figure 11 demonstrates how difficult it can be to distinguish background noise from apoptosing retinal cells (see in particular inside the white dashed circle). The yellow arrows point towards examples of pink spots which were likely to be labeled as cells by the operators. Once again, the small size and low fluorescence of these spots prevented labeling by the algorithm, but also served to prevent mislabeling of noise as cells. Another reason why RGC spots were undercounted by the algorithm was due to the shape of the spots, as shown in Figure 12.

The yellow circle contains spots labeled as non-cellular structure (in pink) by the algorithm, which should have been labeled as RGC spots (in green). This is due the elongated non-circular shape of the spots on the image (resulting from image aberration), which prevents them being labeled as cells by the algorithm (see ‘Methods’ section).

### Analysis of individual manual operator cell counts vs automated cell counts

As shown in Table 1, there is a highly significant Pearson correlation (p < 0.001) between the manual cell counts measured by all three operators, as well as between the automated cell count and each operator’s manual cell count.

**Table 1:** Statistical analysis of the automated cell count and the individual operator cell counts

		**Paired samples correlations**		
**Cell count pair**	**Mean cell count difference**	**Pearson R**	**R**^**2 **^**value**	**Sig. (two-tailed)**
Operator 1 & Automated	13.4	0.974	0.949	0.001
Operator 2 & Automated	0.5	0.976	0.953	0.001
Operator 3 & Automated	−14.7	0.967	0.936	0.001
Operator 1 & Operator 2	12.9	0.984	0.969	0.001
Operator 1 & Operator 3	28.1	0.977	0.955	0.001
Operator 2 & Operator 3	15.2	0.992	0.983	0.001

Within all three manual cell counts and the automated cell counts, the Intraclass correlation coefficient is 0.994 (p < 0.001, 95% CI 0.991 – 0.996). Cronbach’s alpha measure of consistency was 0.986 for operator 1 and automated cell counts, 0.983 for operator 2 and automated cell counts, 0.980 for operator 3 and automated cell counts and 0.986 for the mean manual (all three operators) cell count and automated cell count. The automated cell count was within 1.96 standard deviations from the manual cell counts of operators 1, 2 and 3 in 61 (92.4%), 62 (93.9%) and 63 (95.5%) images analysed respectively. Overall, there was no significant difference between the three operators’ cell counts (ANOVA, p = 0.319). Table 2 below illustrates the strength of agreement between the automated and the manual counts, as well as the inter-operator agreement.

**Table 2:** Bland Altman test of agreement results between automated cell counts and each individual operator’s cell counts, as well as inter-operator agreement

	**Agreement - Bland Altman test**		
**Cell count pair**	**Bias**	**Upper 95%**	**Lower 95%**
Operators Mean & Automated	5.60%	52.40%	−41.30%
Operator 1 & Automated	−17.30%	31.90%	−66.50%
Operator 2 & Automated	16.40%	110.80%	−77.90%
Operator 3 & Automated	34.40%	121.70%	−53.00%
Operator 1 & Operator 2	31.20%	64.90%	−127.30%
Operator 1 & Operator 3	−48.90%	43.30%	−141.10%
Operator 2 & Operator 3	−17.90%	72.70%	−108.60%

The inter-operator 95% limits of agreement were wider than those between the mean manual and automated cell counts, indicating wider inter-operator variability. The 66 DARC images contained an average of 126 cells each. Applying the average discrepancy (bias) of 5.6% between methods, this would result in automated cell count difference of 7 cells, which is not clinically important.

## Discussion

Cell counting has numerous applications in the field of biological imaging [27-30]. Although manual counting by an experienced cell biologist remains the gold standard, this process is time-consuming, monotonous, non-reproducible and subject to bias. The procedure proposed here counts cells in DARC images of variable quality to a level of confidence that is comparable to the gold-standard manual method. This technique has the advantages of being fast, accurate, reproducible and non-labour intensive. Fixing the algorithm parameters before image analysis enabled a non-biased objective quantification of cells that minimises cell count variability arising from inter-observer variability.

Various methods have been developed for automated retinal image analysis [31-34]. Fluorescence images present specific challenges for the development of automated methods of cell counting, particularly the problem of background noise being mislabeled as cells [35]. Distinguishing fluorescent particles from background noise and mild non-specific staining is therefore a crucial step in the development of algorithms enabling automated labeling and counting of fluorescent cells [28]. Increasing the image-thresholding level (without preprocessing) minimizes the impact of noise on cell-counts but results in more fluorescent cells being missed. The pre-processing stage of our algorithm minimises the impact of noise on local image statistics (such as local mean luminance and contrast) allowing us to use lower thresholds and so detect more cells without mislabeling noise.

Fluorescent cells may present as circular regions containing relatively uniform luminance structure, or may be more non-uniform in terms of shape and luminance [35]. Non-uniform cell shape is a common problem in 2D histological sections of 3D specimens, in which cells may be partially present or damaged due to the sectioning process [34]. Uneven luminance commonly occurs due to uneven fluorescent staining [36,37], and the image acquisition process [38]. The latter may also result in local contrast variability, also impeding the accuracy of automated analysis [39]. In the context of fluorescence image analysis, this limits the utility of automated cell enumeration algorithms relying on cell-shape and luminance [40,41]. To surpass these challenges, Byun et al. [37] used Laplacian-of-Gaussian filtering followed by searching for local maxima using cell size and distance between cells for the detection of cell nuclei in immunofluorescent retinal images acquired by confocal microscopy. In comparison to manual counting, their automated technique counted outer nuclei layer (ONL) nuclei with an average error of 3.67% (0–6.07%) and inner nuclear layer (INL) nuclei with an average error of 8.55% (0–13.76%). Accuracy of the technique was compromised in the INL due to variability in nuclei size and shape [37]. Large variability in cell size may indeed limit the accuracy of automated cell enumeration. Our algorithm utilizes a minimum cell size parameter rather than the mean or median cell size for categorization of cells after image pre-processing and thresholding. This has the advantage of maximizing detection of various size cells, (see Figure 1 in ‘Methods’ as an example) yet minimizing detection of noise and any other smaller background structures. This may be problematic in images containing small cells similar in size to background noise, which is why pre-processing is a crucial step for minimizing error in such images. It is possible to add a ‘maximum’ cell size cut-off to our algorithm, but this was not required for DARC images.

Even in normal ‘non-fluorescein’ images, the presence of noise, fluctuating luminance and non-regular cell structure is a recognized barrier to automated retinal image analysis [31,33,42,43]. The algorithm presented here utilized image pre-processing, thresholding and blob analysis to enable detection of non-uniform and irregular fluorescent apoptosing retinal cells from noise, and other non-cellular structures (such as parts of blood vessels). We suggest that our algorithm may be more widely applicable to cell labeling problems in both retinal and other biological images with poor image quality and various shaped structures (e.g. elongated structures such blood vessels or nerves), but this is yet to be tested.

There are no studies we can find which have developed automated techniques for labeling and counting of single apoptosing retinal cells. This limits the comparability of our automated cell detection method to other methods. Barnett et al. have utilized a cell penetrating fluorescent peptide probe (TcapQ) in an *in vivo* rat model of glaucoma to image single apoptosing RGCs by *ex vivo* fluorescence imaging [44]. Counting of the apoptosing retinal cells was computer-assisted; the authors state that quantification of RGCs was performed by Scion image analysis software (Scion Corp), and that an experienced observer (who was blinded to the procedure) performed the counting process. The quantification of RGCs was therefore operator-dependent and not comparable to our automated algorithm. More recently, Qiu X et al. used a confocal scanning laser ophthalmoscope (CSLO) to enable *in vivo* fluorescence imaging of activated apoptosing RGCs displaying TcapQ probe activation [45]. Strong fluorescent cell-specific signals were observed with *in vivo* imaging in the RGC layer of eyes of living rats pre-treated with NMDA followed by TcapQ488. Image analysis was performed manually; cell signals were counted by a human operator using ImageJ software. The authors performed automated cell counting in a ‘subset’ of animals using “Find Maxima” in ImageJ to confirm manual counting. Noise tolerance level was pre-set, while edge and center (optic disc) maxima were excluded from the analysis field. Once again, an accurate and efficient automated method of cell quantification would be of great use in such studies. The evolving ability to image single apoptosing retinal cells *in vivo* and the potential of this technology to be used in humans in the future highlights the need for an accurate method of quantifying apoptosing RGCs that is not operator-dependent.A weakness of the algorithm is that the automated cell counts tended to be lower than the mean manual cell counts for DARC images with RGC counts of >200 cells. Although these cell counts were within 1.96 SD from the mean difference as shown on Figure 7. The two principal factors for RGC spots being mislabeled as non-cellular structures were 1) Elongated non-circular RGC spots (due to image aberration), and 2) small and low luminance spots. For the former, the algorithm could be equipped with a function in which the operator adjusts the minimum aspect ratio for DARC images in which image acquisition has resulted in RGC spots appearing elongated. This has not been tested in this study. As for small and low luminance spots, reducing the cell size cut-off or lowering the luminance threshold may result in more noise being mislabeled as cells. Furthermore, pink spots which have been labeled as cells by operators in Figures 10 and 11 are not clear-cut apoptosing RGC spots, and may be argued to be noise rather than apoptosing cells. It is important to note that overall, the average automated cell count discrepancy was 5.6% higher than the mean manual cell count. The pattern of lower total cell counts obtained by the automated algorithm in images with >200 cells may be due to inadequately sized sample (14 out of 66 DARC images contained >200 cells as per mean manual count). A future comparative study of DARC images with >200 cells will shed more light on this. As DARC is a fairly new technology and still experimental, it is still not established whether such small low luminance spots are cellular or non-cellular. Arguably, only clear-cut RGC spots should be labeled and counted by manual or automated methods to minimize bias. As DARC imaging improves, visualization of small apoptosing RGC will become easier. Furthermore, if this technique succeeds in humans (Human clinical trials due to start soon), apoptosing RGC’s should be larger and easier to identify.

A further weakness of our study is our assumption of the three operators’ mean cell count as a gold-standard apoptosing cell count. In reality, even an experienced operator cannot be assumed to be able to label and count apoptosing retinal cells in DARC images with 100% accuracy, and this method is subjective. The operator needs to be able to distinguish positive-labeled cells, which may be difficult due to the small size of apoptosing retinal cells, the presence of non-specific staining, and the ‘granular’ nature of the retinal background especially apparent in poor quality images. To eliminate any subjective bias in the automated method, a pilot study was performed to determine and preset the optimum minimum cell size cut-off which could be applied to DARC images of variable quality. Furthermore, our comparison of total cell counts may not be the sharpest instrument for looking at relative strengths and weaknesses of operators and algorithms. It is possible to use a more “multi-local” analysis, looking at differences in correspondence of assigned labels *within a locale* to provide a more detailed comparison of manual and automated analysis techniques, and this is an approach we are currently evaluating.

## Conclusion

The novel Matlab software script described in this study enables fast, reproducible and non-operator dependent semi-automated labeling and counting of apoptosing retinal cells. The automated cell counts have significant correlation and consistency with the gold-standard mean manual cell counts, with no significant difference being detected. The method utilises fixed parameters, thus enabling analysis by relatively inexperienced operators. If image cropping and/or re-sizing is needed, it can be incorporated into the Matlab algorithm to make the image analysis process fully automated. This automated technique may prove to be a valuable method of quantifying apoptosing retinal cells, with particular relevance to translation in the clinic, where a Phase I clinical trial of DARC in glaucoma patients is due to start shortly.

## Availability of supporting data

The cell count results of the operators and the automated algorithm are available in the LabArchives repository, [Dataset DOI:10.6070/H4HM56D2 and ‘https://mynotebook.labarchives.com/share/Bizrah/MjAuOHwzNzM4Ny8xNi9UcmVlTm9kZS8zODcyMTExMDMyfDUyLjg’].

## Competing interests

All authors declare that they have no competing interests.

## Authors’ contributions

MB designed the study, collected the data, performed data analysis and interpretation, and wrote the manuscript. SCD wrote the automated algorithm and helped write the manuscript. FR and MP performed manual cell counting and helped design the study. LG, EN, SN generated the DARC images used for analysis and helped design the study. BD and AY helped in statistical data analysis and interpretation, and helped write the manuscript. FC directed the study and helped write the manuscript draft. All authors read and approved the final manuscript draft.
